# Group analysis data representing the effects of frontopolar transcranial direct current stimulation on the default mode network

**DOI:** 10.1016/j.dib.2018.08.164

**Published:** 2018-08-31

**Authors:** Jeesung Ahn, Jonghyun Lee, Jin Hyuk Han, Min Seong Kang, Sanghoon Han

**Affiliations:** a Graduate Program in Cognitive Science, Yonsei University, Seoul 03722, Republic of Korea; b Department of Psychology, Yonsei University, Seoul 03722, Republic of Korea; c Department of Applied Statistics, Yonsei University, Seoul 03722, Republic of Korea

**Keywords:** tDCS, fMRI, Resting-state, Default mode network, Group independent component analysis, Frontopolar prefrontal cortex

## Abstract

The current data provide information about altered activities of the default mode network (DMN) after applying transcranial direct current stimulation (tDCS) over the frontopolar prefrontal cortex. To explore whether frontopolar tDCS with a small current intensity and small electrodes can induce changes in the DMN, resting-state functional magnetic resonance imaging (fMRI) data were collected before and after the application of tDCS. The results of independent component analysis using the resting-state fMRI data are reported in this article.

**Specifications table**

**Table:** 

Subject area	Neuroscience
More specific subject area	Cognitive Neuroscience
Type of data	Table, Figure
How data was acquired	Functional magnetic resonance imaging (fMRI), 3 T Philips using a 32-channel radiofrequency head coil
Data format	Analyzed
Experimental factors	Group: active transcranial direct current stimulation (tDCS) group, sham tDCS group
	Session: pre-tDCS, post-tDCS
Experimental features	Resting-state fMRI data were collected before and after application of tDCS
Data source location	Seoul, Korea
Data accessibility	Data provided in article

**Value of the data**

• The current data provide information about the effects of tDCS on the DMN as an independent component (IC) of resting-state fMRI.
• The data can be used to assess whether current intensity smaller than 0.5 mA and electrodes smaller than a conventional size (5 × 7 cm^2^) can induce alterations of intrinsic functional networks.
• The data can be used to explore the effects of tDCS administered to the bilateral frontopolar prefrontal cortex.
• The data can be compared to other tDCS studies with various electrode placements, electrode sizes, current densities, and total charges. The comparison with other studies can be useful in selecting potential tDCS parameters.

## 1 Data

We collected resting-state fMRI data from 40 participants before and after the application of tDCS. Twenty participants received active tDCS while sham tDCS was applied to the other 20 participants. The data presented here include the information about independent component analysis (ICA) procedures for analyzing resting-state fMRI and results of comparing each group and session using ICs that represent the DMN.

## 2 Experimental design, materials and methods

### 2.1 Participants

A total of 40 healthy adults (16 women, 24 men) with a mean age (*SD)* of 25.6 years (2.8 years) participated in the study for monetary compensation ($25). All participants reported being right-handed, with normal or corrected-to-normal vision and no other contraindications for MRI. All provided written informed consent to participate which was approved by Institutional Review Boards of Yonsei University.

### 2.2 Experimental procedures

Participants were randomly assigned to receive a single 15-min session of anodal (Active tDCS group, *n* = 20, 8 women, 12 men, mean age = 26) or sham tDCS (Sham tDCS group, *n* = 20, 8 women, 12 men, mean age = 25.3). The study was conducted in a single-blind, randomized, and sham-controlled design to guarantee participants were unaware of the stimulation condition. All participants were given the same instruction that they were going to receive active tDCS. An initial scan was conducted before the tDCS procedure to acquire resting-state fMRI at baseline. Fifteen minutes of tDCS was subsequently applied outside the scanner followed by another session of resting-state fMRI. Each run of resting-state fMRI was acquired for 10 min, during which participants were instructed to lie still with eyes closed, relaxed, and not to fall asleep.

### 2.3 tDCS application

A direct current of 0.5 mA for 15 min was induced by a pair of saline-soaked sponge electrodes (3.5 × 3.5 cm^2^) and delivered by a battery-driven, constant-current stimulator (http://www.foc.us; ©FOC.US LABS/EUROPEAN ENGINEERS). The current density was 0.0408 mA/cm^2^, which can be calculated by dividing the current intensity with the surface area of the electrode. The anode was placed above FP1 corresponding to the left frontopolar prefrontal cortex (FPC) according to the 10–20 international system for EEG, and the cathode was positioned over FP2 corresponding to the right FPC [1]. The current at the intensity of 0.05 mA was determined to explore the effects of small current intensity in tDCS. Also, relatively smaller electrodes compared to conventional ones were used in order to constrain the electric field induced by tDCS exclusively to the FPC [2]. The same procedures were applied for the sham tDCS except that tDCS was automatically turned off 30 s after start without noticing participants.

### 2.4 Functional data acquisition

fMRI data were collected on a 3.0 T Philips Ingenia CX MRI scanner equipped with a 32-channel head coil. Each resting-state run included 300 whole-brain volumes acquired using a T2*-weighted single-shot echo-planar imaging (EPI) sequence with the following parameters: repetition time (TR), 2000 ms; echo time (TE), 30 ms; flip angle (FA), 90°; in-plane resolution, 3.75 × 3.75 mm^2^; slice thickness, 4 mm; no slice gap; number of slices, 33; matrix size, 64 × 64; field-of-view (FoV), 240 × 240 mm^2^.

### 2.5 Functional data preprocessing

Functional images were preprocessed using Data Processing Assistant for Resting-State fMRI (DPARSF, version 4.3, http://rfmri.org/DPARSF) toolbox [3]. Preprocessing steps included a slice-timing correction, motion correction, spatial normalization to the Montreal Neurological Institute (MNI) template, resampling into 3 × 3 × 3mm^3^ size voxels, spatial smoothing using a Gaussian kernel with a full width at a half maximum (FWHM) of 4 mm, linear detrending, regressing out nuisance covariates (six head-motion parameters, cerebrospinal fluid and white matter signals), and low-pass filtering with a frequency cut-off of 0.08 Hz.

### 2.6 Independent component analysis

For each subject, spatial ICA was performed on each resting-state run using the software of Group ICA of fMRI Toolbox (GIFT, version 4.0b; http://mialab.mrn.org) run on MATLAB 2017a [4]. Following parameters were used for conducting ICA: the number of output components, 30; ICA algorithm, extended Infomax; back-reconstruction method, spatial-temporal regression. The intensity values of each resulting component were scaled to *z*-score to display which voxels most strongly contributed to constituting a particular IC. A template-matching procedure was performed using a template of the default mode network (DMN) provided in GIFT to select the best-fit component for the DMN from each subject׳s ICA data [5]. The template-matching procedure involved calculating goodness-of-fit (GOF) of an individual spatial component by subtracting the mean *z*-score of voxels outside the DMN template from the mean *z*-score of voxels within the template, and selecting a component with the highest GOF value [6].

**Fig. 1:**
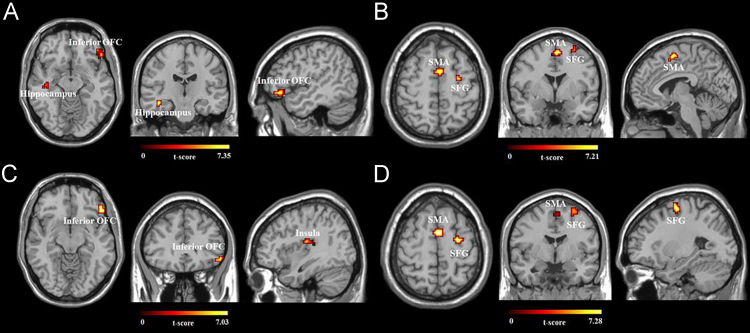
Brain areas playing a different role in the DMN after the application of tDCS (FWE-corrected, *p* < 0.05, *k* > 10 voxels).A.The left hippocampus and right inferior orbitofrontal cortex (OFC) showed reduced coactivity within the DMN after active tDCS compared to sham tDCS.B.Increased coactivation was found in the right supplementary motor area (SMA) and superior frontal gyrus (SFG) after active tDCS compared to sham tDCS.C.The deficient coactivity of the right inferior OFC and right insula after active tDCS mainly contributed to the interaction effect of (A).D.The right SMA and right SFG played more prominent roles in constituting the DMN after active tDCS, which mainly contributed to the interaction effect of (B).

Then, the best-fit ICs representing the DMN were gathered from each subject for a random effect analysis. We used a full-factorial design in Statistical Parametric Mapping (SPM 12, Wellcome Trust Centre for Neuroimaging, University College London) for analysis of variance (ANOVA) to test the interaction of Group as a between-subjects factor (active vs. sham tDCS group) and Session as a within-subjects factor (pre- vs. post-tDCS). Specifically, active tDCS group (pre–post) > sham tDCS group (pre–post) and active tDCS group (post–pre) > sham tDCS group (post–pre) were examined. Furthermore, the full-factorial design allowed us to calculate all possible contrasts of interest as post-hoc tests including pre active tDCS vs. post active tDCS and pre sham tDCS vs. post sham tDCS. For the post-hoc analysis, the voxels that showed the interaction effect between group and session at an uncorrected threshold of *p* < 0.005 were used as a mask. To correct for multiple comparisons, whole-brain family-wise error (FWE) correction was used throughout all analyses (FWE-corrected, *p* < 0.05, *k* > 10 voxels, Table 1 and Fig. 1).

**Table 1:** Brain areas showing different activities within the DMN after the application of tDCS (FWE-corrected, *p* < 0.05, *k* > 10 voxels).

**Regions**	**L/R**			**MNI coordinates**			**t_max_**
				***x***	***y***	***z***	
***Pre Active tDCS – Post Active tDCS > Pre Sham tDCS – Post Sham tDCS***							
Hippocampus		Left	−42		−24	−18	7.35
Inferior Orbitofrontal Cortex		Right	51		30	−12	6.63
***Post Active tDCS – Pre Active tDCS > Post Sham tDCS – Pre Sham tDCS***							
Superior Frontal Gyrus		Right	27		−9	57	6.16
Supplementary Motor Area		Right	3		−3	57	5.77
***Active tDCS Group: Pre tDCS > Post tDCS***							
Inferior Orbitofrontal Cortex		Right	54		30	−12	7.03
Insula		Left	−36		−18	15	5.84
Superior Temporal Gyrus		Left	−39		−30	12	5.30
***Active tDCS Group: Post tDCS > Pre tDCS***							
Superior Frontal Gyrus		Right	27		−9	57	7.21
Supplementary Motor Area		Right	3		−3	57	6.65
***Sham tDCS group: Pre tDCS > Post tDCS***							
No suprathreshold clusters							
***Sham tDCS group: Post tDCS > Pre tDCS***							
No suprathreshold clusters
